# Cross-Country Comparison of Public Awareness, Rumors, and Behavioral Responses to the COVID-19 Epidemic: Infodemiology Study

**DOI:** 10.2196/21143

**Published:** 2020-08-03

**Authors:** Zhiyuan Hou, Fanxing Du, Xinyu Zhou, Hao Jiang, Sam Martin, Heidi Larson, Leesa Lin

**Affiliations:** 1 School of Public Health Fudan University Shanghai China; 2 Global Health Institute NHC Key Laboratory of Health Technology Assessment Fudan University Shanghai China; 3 Centre for Clinical Vaccinology and Tropical Medicine University of Oxford Oxford United Kingdom; 4 Department of Infectious Disease Epidemiology London School of Hygiene & Tropical Medicine London United Kingdom; 5 Department of Health Metrics Sciences University of Washington Seattle, WA United States

**Keywords:** COVID-19, internet, surveillance, infodemic, infodemiology, infoveillance, Google Trends, public response, behavior, rumor, trend

## Abstract

**Background:**

Understanding public behavioral responses to the coronavirus disease (COVID-19) epidemic and the accompanying infodemic is crucial to controlling the epidemic.

**Objective:**

The aim of this study was to assess real-time public awareness and behavioral responses to the COVID-19 epidemic across 12 selected countries.

**Methods:**

Internet surveillance was used to collect real-time data from the general public to assess public awareness and rumors (China: Baidu; worldwide: Google Trends) and behavior responses (China: Ali Index; worldwide: Google Shopping). These indices measured the daily number of searches or purchases and were compared with the numbers of daily COVID-19 cases. The trend comparisons across selected countries were observed from December 1, 2019 (prepandemic baseline) to April 11, 2020 (at least one month after the governments of selected countries took actions for the pandemic).

**Results:**

We identified missed windows of opportunity for early epidemic control in 12 countries, when public awareness was very low despite the emerging epidemic. China's epidemic and the declaration of a public health emergency of international concern did not prompt a worldwide public reaction to adopt health-protective measures; instead, most countries and regions only responded to the epidemic after their own case counts increased. Rumors and misinformation led to a surge of sales in herbal remedies in China and antimalarial drugs worldwide, and timely clarification of rumors mitigated the rush to purchase unproven remedies.

**Conclusions:**

Our comparative study highlights the urgent need for international coordination to promote mutual learning about epidemic characteristics and effective control measures as well as to trigger early and timely responses in individual countries. Early release of official guidelines and timely clarification of rumors led by governments are necessary to guide the public to take rational action.

## Introduction

In early December 2019, the then-unnamed novel coronavirus disease (COVID-19) emerged in Wuhan City and spread rapidly across China [1,2]. On January 23, 2020, the Chinese government placed Wuhan and several nearby cities under quarantine and implemented containment measures to slow community transmission [3]. The World Health Organization (WHO) declared the outbreak to be a public health emergency of international concern (PHEIC) on January 30, when there were almost 8000 confirmed cases worldwide; all but 98 of these cases, along with all 170 COVID-19–related deaths, were in China. The PHEIC declaration stressed the risk the virus posed to countries beyond China and the need for a more coordinated international response to the outbreak. On March 11, the WHO declared COVID-19 a global pandemic, with a worldwide confirmed case count of 118,000 in 114 countries and the death toll reaching more than 4200. At that time, more than 90% of cases were localized in four countries, including China and South Korea, where local epidemics had significantly declined [4]. By the end of April, the epidemic had spread to 213 countries with more than 3.02 million confirmed cases, leading to at least 208,000 deaths [5]. The United States reported the highest numbers of confirmed cases and deaths, followed by Spain, Italy, the United Kingdom, Germany, and France; all these countries reported over 120,000 cases and 24,000 deaths except for Germany, which reported approximately 6000 deaths [5].

During an epidemic, it is crucial to understand how critical information about the health threat is disseminated and how the public processes and responds to this information [6,7]. The risks and uncertainties of emerging infectious diseases may arouse public awareness and prompt either constructive behavior (eg, employing personal hand hygiene and avoiding mass gatherings) or disruptive behavior (eg, panic buying and adopting unproven treatments) [7,8]. COVID-19 has triggered the spread of rumors and misinformation through social media regarding unproven remedies, which has induced public stress and panic [9,10]. In addition, the public may respond differently to an epidemic across countries. In the early stage of the COVID-19 epidemic, people in Asian countries immediately began to wear face masks; however, Europeans and North Americans opposed this practice [11]. Messages from health authorities and evidence of the effectiveness of masks against COVID-19 are conflicting [12]. It is necessary to understand why and how the public responds to COVID-19–related information, which will further inform government risk communication and appropriate official guidelines [13].

The power of internet search data is being increasingly recognized in public health emergencies [14,15]. In contrast with traditional surveys, internet surveillance can systematically track public responses to epidemics in real time and is less likely to be affected by recall bias [16]. Despite these benefits, the role of internet surveillance (also called infoveillance or infodemiology) in monitoring public behavioral responses and rumors during an epidemic is still underexplored [17-19]. Using internet surveillance data from China and worldwide, this study aimed to assess the public awareness and behavioral responses in real time during the first 100 days of the COVID-19 epidemic. This study compares the governmental and public responses across selected countries and provides insights on the control of COVID-19 and future epidemics.

## Methods

### Study Setting

In this study, we conducted internet surveillance in 12 countries. In addition to China, three countries in East and Southeast Asia that were affected by the first wave of the pandemic were selected, namely Japan, South Korea, and Singapore, followed by four European countries (Italy, France, Spain, and the United Kingdom) and the United States. Additionally, Brazil, South Africa, and India, where internet surveillance data (Google Trends) are available, were selected from Latin America, Africa, and South Asia, respectively. All data are publicly available.

### Data Collection

Internet surveillance was used to collect real-time data from the general public to assess public awareness and rumors (China: Baidu; worldwide: Google Trends) and behavior responses (China: Ali Index; worldwide: Google Shopping). The data cover the period from December 1, 2019 (prepandemic baseline) to April 11, 2020 (at least one month after the governments of selected countries took actions to address the COVID-19 pandemic). Table 1 lists the keywords used to measure public awareness, rumors, and behavioral responses to the COVID-19 epidemic.

**Table 1:** Keywords searched for public awareness, behavioral responses, and rumors regarding the COVID-19 epidemic.

Domain			Keywords
Awareness of COVID-19^a^			*coronavirus* (冠状病毒), *Wuhan pneumonia* (武汉肺炎)
Behavioral response to protection measures			*mask* (口罩), *hand sanitizer* (洗手液), *disinfectant* (消毒液/消毒剂/消毒水), *thermometer* (体温计)
**Rumors**			
	Worldwide	*chloroquine*, *hydroxychloroquine*	
	China	*radix isatidis* (板蓝根), *Shuanghuanglian* (双黄连), *garlic* (大蒜)	

^a^COVID-19: coronavirus disease.

#### Google Trends

Google Trends can provide insight into the relative search volumes of search terms on Google on a daily basis [20]. Depending on the source of the search, Google Trends can be further divided into web search trends and Google Shopping trends, and these trends were highly correlated (Multimedia Appendix 1). The Google Trends index (web search) on the topic of *coronavirus* was used to assess the awareness of COVID-19 among the general public, whereas the Google Shopping indices on two topics, *mask* and *hand sanitizer*, were used to assess the adoption of personal protection measures. In countries where Google Shopping data were limited for assessing public purchasing behavior (including Japan, Singapore, South Korea, Italy, and Spain), Google web search data were used as an alternative. Google Trends presents relative search volumes ranging from 0 to 100 (the maximum daily search volume on specific terms is standardized as 100%).

The antimalarial drugs chloroquine and hydroxychloroquine have been repeatedly mentioned by world-leading politicians as treatments for COVID-19 without clinical data to support their efficacy; these drugs have potentially deadly side effects [21,22]. By analyzing Google Trends data on both drugs, we assessed the public behavioral response towards misinformation worldwide.

#### Baidu Index

The Baidu search engine, which is Google’s equivalent in China, has more than 1 billion Chinese users. The Baidu Index, which stems from the frequency of searches using the Baidu search engine, is powered by Baidu statistics and exhibited as preset keywords. The Baidu Index reflects the daily number of searches on specific keywords, thereby assessing public awareness and intended behavior. We manually scanned and identified all Baidu Index keywords that included coronavirus, recommended personal protection measures, and rumors and misinformation. For comparison, we also gathered Baidu Index data from the same time period in the previous year as the baseline: December 1, 2018, to April 11, 2019.

Rumors and misinformation circulated widely in China regarding certain herbal medicines for personal health protection. From the Sina Weibo “Hot Search” ranking, we identified three keywords related to these rumors and misinformation: *radix isatidis*, *Shuanghuanglian*, and *garlic*. The Baidu Index on these terms was used to detect public behavioral responses regarding rumors and misinformation in China.

#### Ali Index

The Ali platform, Amazon’s equivalent in China, is a powerful and popular web-based electronic commerce marketplace with more than one billion users, and its purchasing index reflects the number of specific products purchased on the internet. The Ali Index was used to collect behavioral data during the epidemic. The National Health Commission of China issued a series of guidelines recommending that residents adopt personal protection measures consisting of four main aspects: respiratory protection, hand hygiene, home disinfection, and health monitoring [3]. Accordingly, we identified *mask*, *hand sanitizer*, *disinfectant*, and *thermometer* as keywords to reflect the above four measures. We employed these four keywords relevant to recommended personal protection measures to assess public behavioral responses to the COVID-19 epidemic (the Ali platform did not generate an index for rumor-related items).

### Data Analysis

Public awareness on the epidemic was assessed using the Baidu daily indices in China and Google Trends indices worldwide. We assessed the intentions (Baidu Index) and behaviors (Ali Index) regarding the adoption of recommended personal protection measures as well as rumors about ineffective treatments in China; Spearman rank correlation analyses between the Baidu and Ali indices were employed to detect the consistency of intention and behaviors. Google Trends was used to assess the intended behavior regarding personal protection measures and antimalarial drugs worldwide. These indices were used to measure the daily number of searches or purchases related to specific keywords, which we then compared with daily data on numbers of confirmed COVID-19 cases. The Spearman rank correlation was used to examine the correlations between Google Trends for *mask* and *hand sanitizer* and the numbers of new COVID-19 cases in the selected countries. The indices were input into charts to observe the trends along the period of observation and facilitate the trend comparisons across countries.

## Results

### Public Awareness and Searches Related to COVID-19

**Figure 1:**
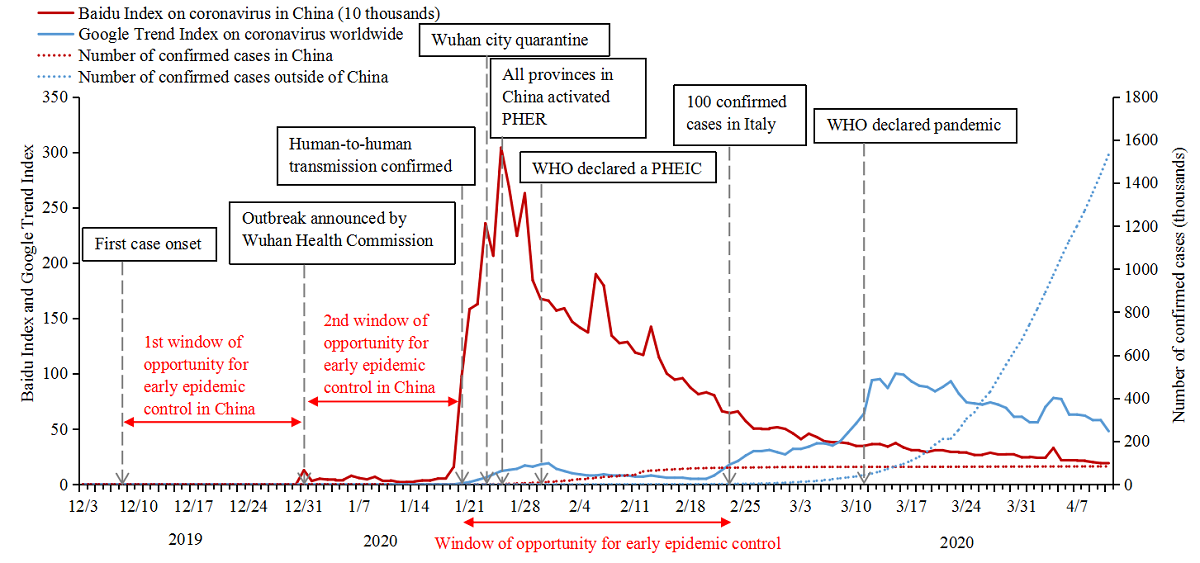
Public awareness and searches related to COVID-19 by Baidu Index in China and Google Trends worldwide from December 2019 to April 2020. PHEIC: public health emergency of international concern; PHER: public health emergency response.

The first COVID-19 case emerged on December 8, 2019, and it was first announced by the Wuhan Health Commission on December 31. As shown in Figure 1, the Baidu search index for *coronavirus* showed no results throughout most of December but jumped to 127,336 searches on December 31, 2019, when the Wuhan Health Commission first reported 27 patients with pneumonia of unknown cause. However, this number immediately decreased to 30,504 searches the next day and remained low between January 1 and 19, 2020. Starting on January 20, when the National Health Commission of China confirmed human-to-human transmission, this number increased sharply, and it peaked at 3,039,324 on January 25. Wuhan City implemented a series of quarantine measures starting January 23, followed by 30 provinces (autonomous regions and municipalities) across mainland China (excluding Tibet). After that, public searches steadily decreased with minor fluctuations. Figure 1 highlights two missed windows of opportunity for early epidemic control in China.

Outside of China, although there was a slight increase in public searches following the confirmation of human-to-human transmission of COVID-19, the Google Trends index for *coronavirus* began to decline on January 31, the day after the WHO declared the outbreak a PHEIC. This index continued to decline and remained at a low level until late February, when COVID-19 started to spread in Italy. Figure 1 shows that the world missed an additional 1-month window of opportunity for early epidemic control, even when the number of COVID-19 cases had reached almost 80,000 in China.

### Behavioral Responses of Adopting Recommended Personal Protection Measures

**Figure 2:**
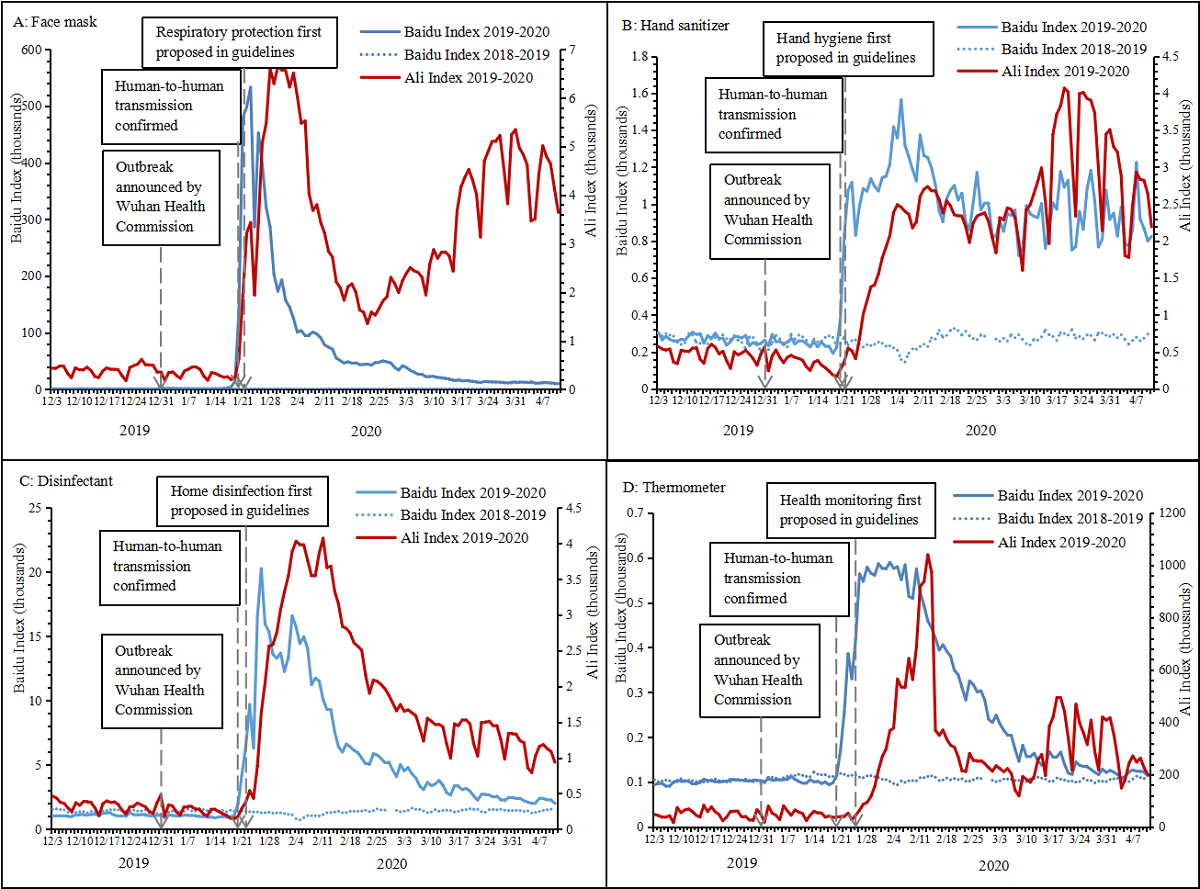
Trends of Baidu and Ali indices for recommended personal protection measures in China from December 2019 to April 2020: A. Face mask. B. Hand sanitizer. C. Disinfectant. D. Thermometer.

The National Health Commission of China first proposed respiratory protection and hand hygiene as protection guidelines on January 21, 2020, and the use of home disinfection and health monitoring on January 22 and 25, respectively [3]. As shown in Figure 2, the Baidu and Ali indices of the four recommended personal protection measures, except for the Ali Index of *thermometer*, all started to increase sharply on January 21, with an exceptional drop on January 24 (Chinese New Year's Eve). The Ali Index of *thermometer* increased rapidly from January 25. The Baidu Index, which indicated an intention to adopt all four measures, increased earlier than the actual behaviors presented in the Ali Index. Both indices remained steadily low during the same period in the previous year (baseline). These results indicate that the Chinese public responded quickly to the issuing of specific guidelines, and both intended and actual purchasing behavior dramatically increased accordingly. Correlation analysis (Multimedia Appendix 2) showed strong positive correlations between the Baidu and Ali indices for all recommended measures (correlation coefficient range of 0.676-0.860, *P*<.001).

**Figure 3:**
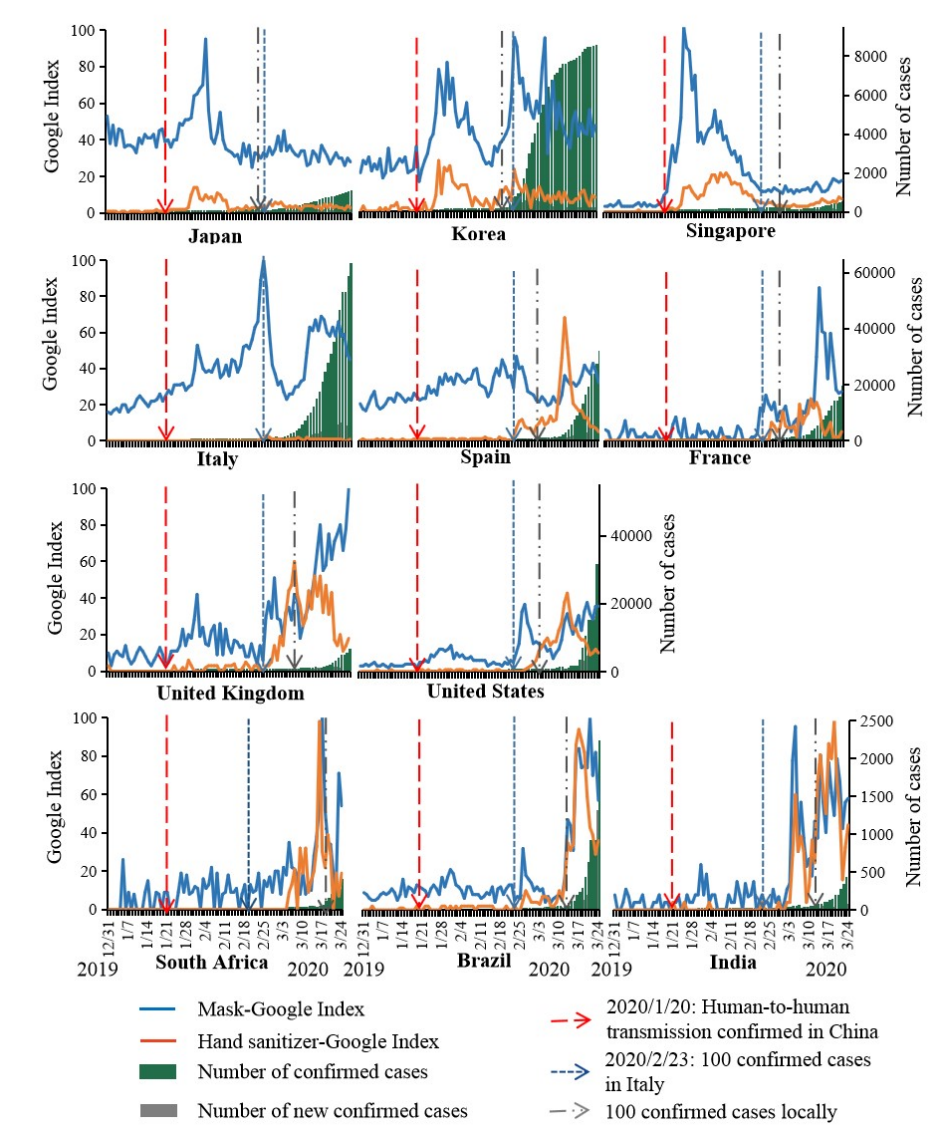
Cross-country comparison of Google Trends data for *mask* and *hand sanitizer* with numbers of COVID-19 cases from December 2019 to April 2020.

Figure 3 presents a cross-country comparison of Google Trends on mask and hand sanitizer with numbers of COVID-19 cases, and their Spearman rank correlation analyses are shown in Multimedia Appendix 3. All listed countries, except for Brazil and South Africa, started to report COVID-19 cases in late January 2020, whereas Brazil and South Africa reported their first cases on February 26 and March 5, respectively. In Japan, South Korea, and Singapore, Google Trends indices on mask and hand sanitizer quickly increased after human-to-human transmission in China and reached a peak around the end of January. The Google indices in Japan and Singapore then decreased and remained steady while the number of COVID-19 cases in these countries was small; meanwhile, with the rapid increase in the number of cases in South Korea, the Google index sharply increased and reached its second peak in late February. Correlation analysis (Multimedia Appendix 3) showed that the Google Trends for *mask* and *hand sanitizer* in the three Asian countries were significantly correlated with new COVID-19 cases in China rather than local cases.

In addition, the Google indices in European countries and the United States remained low after human-to-human transmission in China and started to increase when the number of COVID-19 cases increased from 11 to 123 in Italy on February 23 (Figure 3). In Italy and Spain, where the epidemic first spread among European countries, the Google Trends data for *hand sanitizer* were highly correlated with the number of local cases, whereas the purchase of masks lagged behind the epidemic for 2 to 3 weeks (Multimedia Appendix 3 and Multimedia Appendix 4). The public in India, Brazil, and South Africa did not respond to the spread of COVID-19 in China and Italy or to the PHEIC, with their Google indices remaining low until early March. Even if there was a small increase in the public response to the epidemic in other countries, the index then quickly decreased and did not start to rise again until the epidemic spread to those countries. Overall, the Google Trends data for *mask* and *hand sanitizer* were in line with the trends of the number of cases in each country (Figure 3 and Multimedia Appendix 3), and these trends reached their own peaks when COVID-19 spread locally. From early April, people around the world started to search for and buy face masks.

### Public Responses to Rumors and Misinformation on Remedies

**Figure 4:**
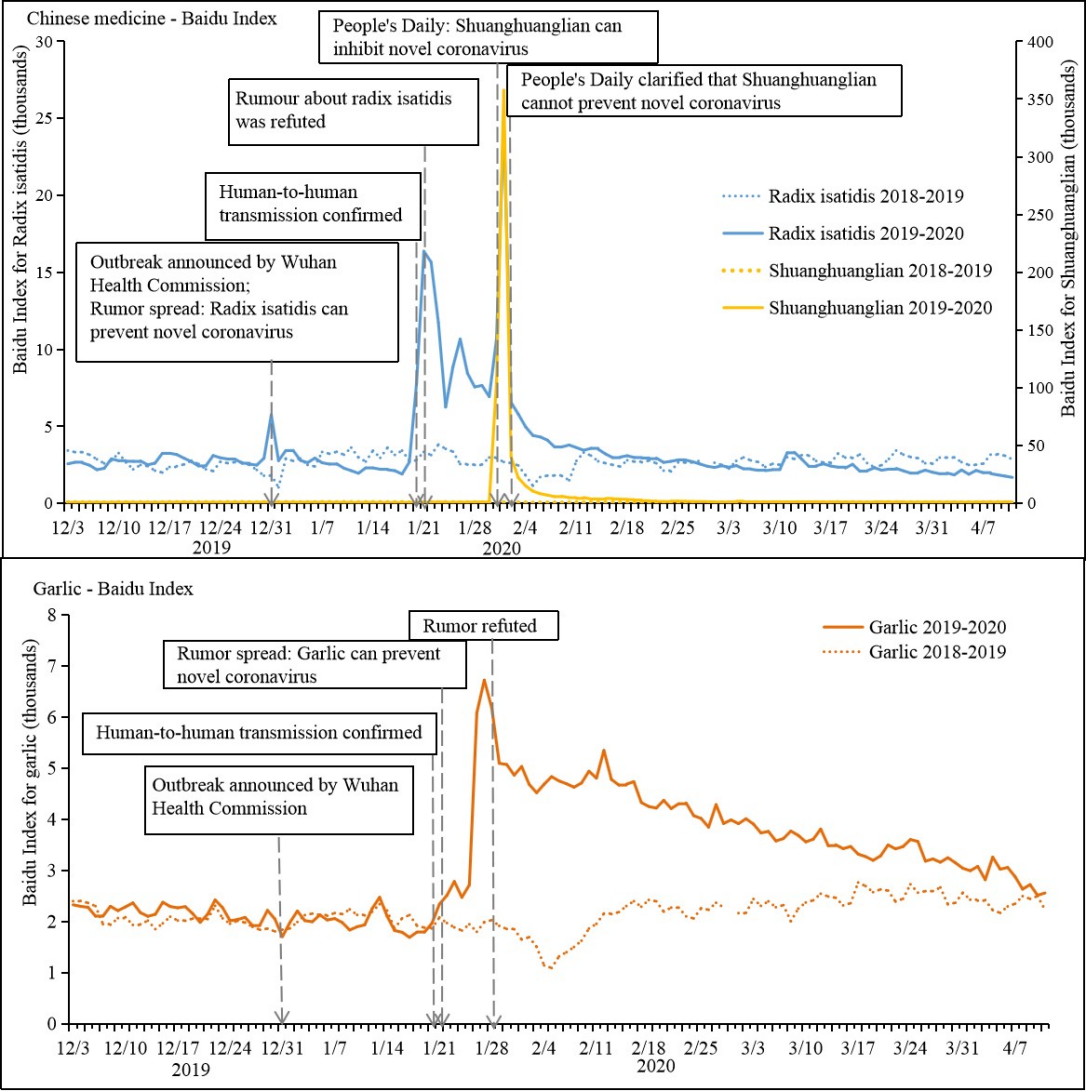
Trends of Baidu indices for rumors related to herbal remedies in China from December 2019 to April 2020.

Figure 4 shows the trends in the Baidu indices on rumors, indicating intended behavior. The Baidu Index of *radix isatidis*, a traditional Chinese medicine used to treat fever, started to increase when the outbreak was first announced. It further increased sharply on January 20, 2020, when human-to-human transmission of COVID-19 and 291 cases were confirmed, and it reached a peak on January 21. The index started to decline from January 21 to 24, during which the newspaper *People's Daily* issued three reports in an attempt to refute rumors of the effectiveness of radix isatidis against COVID-19. On January 31, *People's Daily* reported that Shuanghuanglian, a Chinese medicine, could inhibit COVID-19, and the Baidu indices of both *Shuanghuanglian* and *radix isatidis* rapidly reached their peaks. Both indices decreased rapidly on February 2, when *People's Daily* clarified that Shuanghuanglian cannot prevent COVID-19. A rumor that garlic could prevent COVID-19 started to spread on January 21, and the Baidu Index of *garlic* increased accordingly. This index reached a peak on January 27 and declined after January 28, when *People's Daily* first refuted rumors about the protective function of garlic. Representing the baseline, these indices remained steadily low during the same period in the previous year.

**Figure 5:**
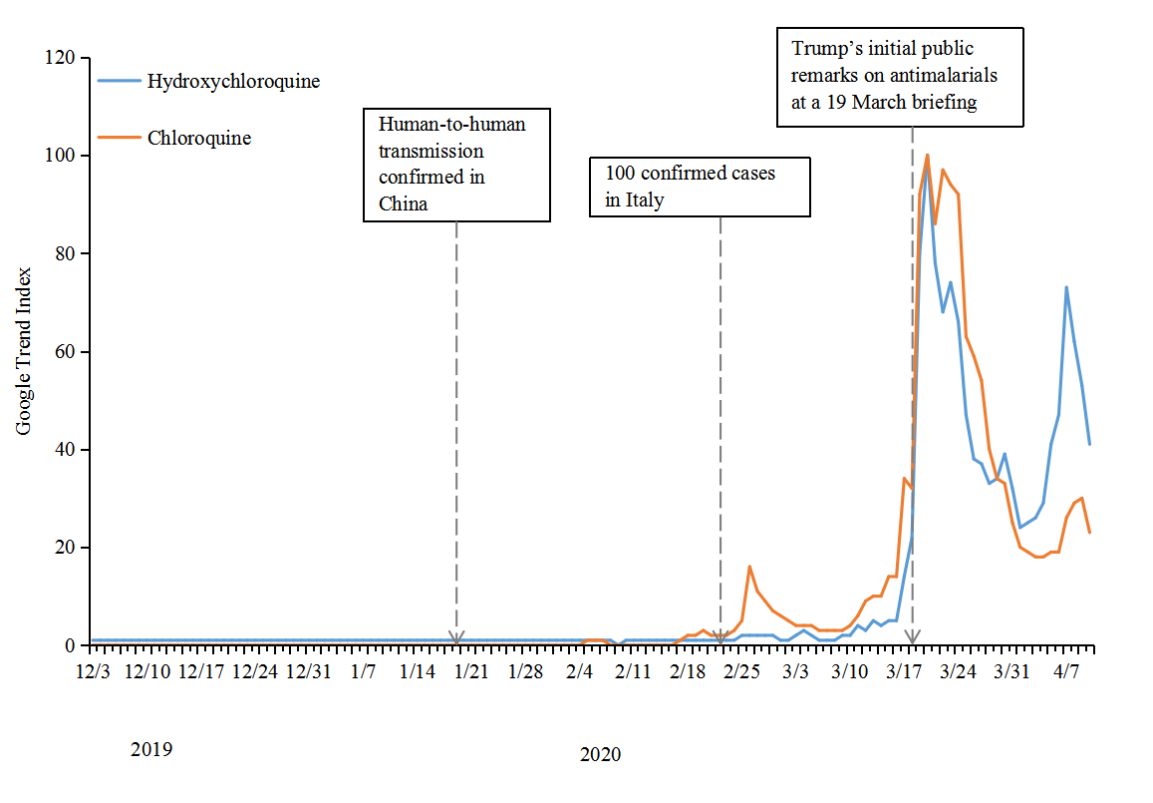
Google Trends data on rumors related to antimalarial drugs worldwide from December 2019 to April 2020.

Figure 5 shows the Google Trends data for the antimalarial drugs chloroquine and hydroxychloroquine worldwide. Public searches for *chloroquine* started to increase and reached a small peak in late February, when COVID-19 started to spread in Italy. The increase in searches for *chloroquine* was observed during this time only in Asian and European countries (Japan, South Korea, Singapore, Italy, France, Spain, and the United Kingdom); it was not found in the United States, India, Brazil, or South Africa. Public searches on both drugs increased after mid-March and quickly peaked on March 20 after US President Donald Trump’s initial public remarks on the drugs at a briefing on March 19; this increase was observed in all countries.

## Discussion

### Principal Findings

Using internet surveillance data, we conducted a cross-country comparison of real-time public awareness and behavioral responses to epidemic information during the first 100 days of the COVID-19 pandemic. We identified squandered windows of opportunity for early epidemic control in 12 countries. The epidemic in China and the PHEIC did not prompt a worldwide public reaction to adopt public health protective measures; instead, most countries and regions only responded to the epidemic after their own case counts mounted. Even if there was a worldwide reaction, the public response to the epidemic in other countries would quickly fall without government leadership and communication. The public responded quickly to official announcements and adopted personal protection behaviors such as buying hand sanitizers; however, rumors and misinformation were found to have led to a surge in sales of herbal remedies in China and antimalarial drugs worldwide. Chinese data showed that the timely governmental clarification of rumors mitigated the rush to buy unproven remedies to treat or prevent COVID-19. This comparative study highlighted the importance of governmental leadership and international coordination in directing epidemic control.

The lack of transparent, timely, and effective risk communication by health authorities around an emerging infectious disease in its early stages failed to bring about appropriate levels of public awareness and behavioral responses, such as avoidance of mass gatherings and personal protection in China, Europe, and the United States. In China, the government did not provide actionable advice for personal protection until January 21. There were two missed windows of opportunity for early epidemic control in China. First, the first COVID-19 case emerged on December 8, 2019, more than three weeks before December 31, when the outbreak was first announced; second, between December 31, 2019, and January 19, 2020, the Wuhan Health Commission made four public announcements with no obvious evidence of human-to-human transmission [1-3]. This series of government announcements kept public awareness low, which prevented the public from realizing the risk of the disease and from taking personal protection measures. This period overlapped with the celebration of the arrival of 2020 and the preparation for the Chinese New Year (January 25), when public attention centered around family reunions. Similarly, our data show an additional window of 4 to 8 weeks of missed opportunity for early epidemic control in Europe and the United States between the PHEIC declaration in late January and the outbreaks in Europe and the United States in March. Transmission of COVID-19 appears to be possible even among people who show no symptoms of the disease; thus, travel restrictions have limited impact on stemming the spread. However, other than flight cancellations and evacuation from China in late January, governments in Europe and the United States did not implement epidemic prevention or control measures such as testing, surveillance, or contact/case tracing [23,24]. Despite reports of local transmissions, population movement between Italy and the rest of the world and public gatherings in these countries were largely unchecked even after the number of cases surged in Italy [25]. Additionally, some political leaders and media outlets repeatedly called the COVID-19 pandemic a hoax [26] and downplayed the threat the pandemic posed to society, leading to a delay in public response.

Further, due to variations in societal and cultural paradigms, face masks are commonly used as a hygienic practice in many Asian countries but are only used by the unwell in European and North American countries [11]. In these countries, authorities discouraged citizens from using face masks, and local communities reacted with stigmatization and racial aggravations against members of East Asian communities who wore masks [12]. Emerging evidence about the efficacy of wearing face masks (“community transmission might be reduced if everyone, including people who have been infected but are asymptomatic and contagious, wear face masks” [11]) finally shifted public opinion. In April 2020, governments in Europe and the United States changed their mask-wearing guidelines and mandated universal face mask use outdoors until an effective vaccine becomes available. Our data show that the use of masks increased substantially after local COVID-19 epidemics began. The sudden shift of government guidelines led to a worldwide surge in the demand for face masks, which have been in severely short supply since the beginning of the epidemic, even for frontline health care professionals. This phenomenon highlights the importance of governmental response in early epidemic preparedness for mobilization and surge capacity, effective control measures, and timely and clear communication to cue public action. Future research is needed to evaluate the impact of mask-wearing policies on awareness and behaviors.

Evidence has proven that early implementation of containment measures can effectively control the COVID-19 epidemic [27]. However, the epidemic in China only caught the public attention of East and Southeast Asia, and the lessons learned in this region did not trigger appropriate epidemic responses in the rest of the world. Similarly, the epidemic in Italy only resulted in public awareness and reaction in Europe and the United States. Due to the dense populations and fragile health systems in South Asian, Latin American, and African regions, COVID-19 spread is of great concern in these countries [28]. This study indicates a need for strengthened international partnerships and coordination to combat the COVID-19 epidemic and future epidemics. The WHO should be empowered to take a leading role in guiding more preparedness actions than solely making statements that a disease constitutes a PHEIC or pandemic.

Our data showed that the public is highly responsive to governmental risk communication during epidemics, suggesting a need for real-time media surveillance. The early release of official guidelines by China’s National Health Commission was effective in guiding the Chinese public to take personal protection measures; however, rumors also triggered several incidents of panic buying (eg, masks, Shuanghuanglian, and garlic), which were quickly calmed after official clarifications. Likewise, references to antimalarial drugs by US President Donald Trump triggered panic searches worldwide. Governments should engage early in public risk communication about epidemic control, detect misinformation, confront inaccurate messages, and clarify rumors in real time [29,30]. Working with the private sector to ensure sufficient stock and reasonable pricing for recommended personal protection products, such as hand sanitizer and masks, is critical for effective epidemic prevention and control and to avoid public panic.

Compared with traditional surveys, internet surveillance tools provide real-time, longitudinal, and dynamic data for capturing public awareness, rumors, and behavioral reactions, and they can be an effective means to evaluate public response towards epidemic information and rumors [19].

### Limitations

This study has several limitations. First, Google Trends are not applicable in all countries, including China and most countries in Africa; therefore, we could only compare countries with available Google Trends data. For countries in which the volumes of Google Shopping data were low (Japan, Singapore, South Korea, Italy, and Spain), we used Google web search data instead. This may have led to inconsistency in the measurements. Second, there is an inherent bias in internet data because the population may be skewed towards younger people. Moreover, Google Trends data related to personal protection measures represent intended behaviors instead of real shopping behaviors. Third, we are unable to conduct segmentation analysis these data to inform audience-tailored communication strategies.

### Conclusion

Our comparative study identified missed windows of opportunity for early epidemic control in 12 countries; it also highlighted the urgent need for international coordination to promote mutual learning regarding epidemic characteristics as well as effective control measures and to trigger early and timely responses in individual countries. During an epidemic, early release of official guidelines and timely governmental clarification of rumors are necessary to guide the public to make rational responses.

## Appendix

Multimedia Appendix 1 Correlations between Google Trends and Google Shopping indices for behavioral responses.

Multimedia Appendix 2 Correlations between Baidu and Ali indices for behavioral responses in China.

Multimedia Appendix 3 Correlations between Google Trends data on the search terms "mask" and "hand sanitizer" and the numbers of new COVID-19 cases.

Multimedia Appendix 4 Lag correlations between Google Trends dada on the search term "mask" and numbers of new COVID-19 cases in Italy and Spain.

## Abbreviations

COVID-19: coronavirus disease
PHEIC: public health emergency of international concern
WHO: World Health Organization
