# Supplementary material for: Cross-Country Comparison of Public Awareness, Rumors, and Behavioral Responses to the COVID-19 Epidemic: Infodemiology Study
Source: J Med Internet Res. 2020 Aug 3;22(8):e21143. doi: 10.2196/21143 (PMC7402643; doi:10.2196/21143)
Supplement: Multimedia Appendix 1 [file jmir_v22i8e21143_app1.docx]

Appendix Table 1: The correlations between Google Trend and Shopping Indices for behavioural response

| Countries | Face mask | | Hand sanitizer | |
| --- | --- | --- | --- | --- |
|  | Correlation coefficient | *P-values* | Correlation coefficient | *P-values* |
| France | 0.855 | <.001 | 0.909 | <.001 |
| The UK | 0.938 | <.001 | 0.853 | <.001 |
| The United States | 0.962 | <.001 | 0.812 | <.001 |
| Brazil | 0.680 | <.001 | 0.832 | <.001 |
| South Africa | 0.654 | <.001 | 0.771 | <.001 |
| India | 0.795 | <.001 | 0.717 | <.001 |
