# Supplementary material for: Cross-Country Comparison of Public Awareness, Rumors, and Behavioral Responses to the COVID-19 Epidemic: Infodemiology Study
Source: J Med Internet Res. 2020 Aug 3;22(8):e21143. doi: 10.2196/21143 (PMC7402643; doi:10.2196/21143)
Supplement: Multimedia Appendix 2 [file jmir_v22i8e21143_app2.docx]

Appendix Table 2: The correlations between Baidu and Ali indices for behavioural response in China

| Recommended protection measures | Correlation coefficient between Baidu and Ali indices | *P-values* |
| --- | --- | --- |
| Face mask | 0.676 | <.001 |
| Hand sanitizer | 0.860 | <.001 |
| Disinfectant | 0.809 | <.001 |
| Thermometer | 0.716 | <.001 |
