# Supplementary material for: Cross-Country Comparison of Public Awareness, Rumors, and Behavioral Responses to the COVID-19 Epidemic: Infodemiology Study
Source: J Med Internet Res. 2020 Aug 3;22(8):e21143. doi: 10.2196/21143 (PMC7402643; doi:10.2196/21143)
Supplement: Multimedia Appendix 3 [file jmir_v22i8e21143_app3.docx]

Appendix Table 3: The correlations between Google Trends on mask/hand sanitizer and numbers of new COVID-19 cases

|  |  | Correlation with COVID-19 cases in each country | | Correlation with COVID-19 cases in China | |
| --- | --- | --- | --- | --- | --- |
| Country | Google Trends | Correlation coefficient | *P-value* | correlation coefficient | *P-value* |
| China | Mask | 0.358 | **0.002** |  |  |
|  | Hand Sanitizer | 0.038 | 0.057 |  |  |
| Japan | Mask | - 0.522 | **<.001** | 0.392 | **<.001** |
|  | Hand Sanitizer | -0.032 | 0.795 | 0.407 | **<.001** |
| Korea | Mask | 0.495 | **<.001** | 0.040 | 0.745 |
|  | Hand Sanitizer | 0.129 | 0.306 | 0.410 | **<.001** |
| Singapore | Mask | -0.209 | 0.103 | 0.798 | **<.001** |
|  | Hand Sanitizer | 0.004 | 0.976 | 0.740 | **<.001** |
| Italy | Mask | 0.240 | 0.081 |  |  |
|  | Hand Sanitizer | 0.632 | **<.001** |  |  |
| Spain | Mask | -0.065 | 0.643 |  |  |
|  | Hand Sanitizer | 0.750 | **<.001** |  |  |
| France | Mask | 0.745 | **<.001** |  |  |
|  | Hand Sanitizer | 0.756 | **<.001** |  |  |
| UK | Mask | 0.774 | **<.001** |  |  |
|  | Hand Sanitizer | 0.708 | **<.001** |  |  |
| US | Mask | 0.759 | **<.001** |  |  |
|  | Hand Sanitizer | 0.798 | **<.001** |  |  |
| South Africa | Mask | 0.310 | 0.183 |  |  |
|  | Hand Sanitizer | -0.027 | 0.911 |  |  |
| Brazil | Mask | 0.733 | **<.001** |  |  |
|  | Hand Sanitizer | 0.727 | **<.001** |  |  |
| India | Mask | 0.751 | **<.001** |  |  |
|  | Hand Sanitizer | 0.846 | **<.001** |  |  |
