# Supplementary figures and images for: Cross-Country Comparison of Public Awareness, Rumors, and Behavioral Responses to the COVID-19 Epidemic: Infodemiology Study
Source: J Med Internet Res. 2020 Aug 3;22(8):e21143. doi: 10.2196/21143 (PMC7402643; doi:10.2196/21143)

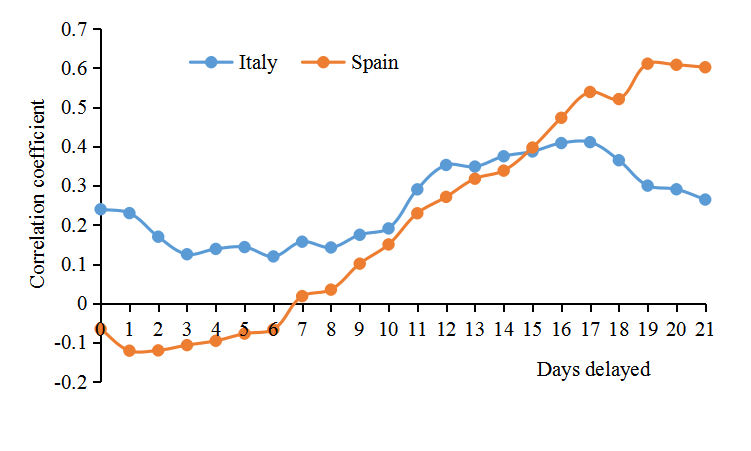

Supplement: Multimedia Appendix 4 [file jmir_v22i8e21143_app4.png]
